# Profiling the Course of Resolving vs. Persistent Inflammation in Human Monocytes: The Role of IL-1 Family Molecules

**DOI:** 10.3389/fimmu.2020.01426

**Published:** 2020-07-10

**Authors:** Paola Italiani, Ettore Mosca, Giacomo Della Camera, Daniela Melillo, Paola Migliorini, Luciano Milanesi, Diana Boraschi

**Affiliations:** ^1^Institute of Protein Biochemistry and Cell Biology, National Research Council, Naples, Italy; ^2^Institute of Biomedical Technologies, National Research Council, Segrate, Italy; ^3^Clinical Immunology Unit, Department Clinical and Experimental Medicine, University of Pisa, Pisa, Italy

**Keywords:** inflammation, monocytes, macrophages, IL-1 family, *in vitro* model

## Abstract

Monocytes and macrophages have a central role in all phases of an inflammatory reaction. To understanding the regulation of monocyte activation during a physiological or pathological inflammation, we propose two *in vitro* models that recapitulate the different phases of the reaction (recruitment, initiation, development, and resolution vs. persistence of inflammation), based on human primary blood monocytes exposed to sequential modifications of microenvironmental conditions. These models exclusively describe the functional development of blood-derived monocytes that first enter an inflammatory site. All reaction phases were profiled by RNA-Seq, and the two models were validated by studying the modulation of IL-1 family members. Genes were differentially modulated, and distinct clusters were identified during the various phases of inflammation. Pathway analysis revealed that both models were enriched in pathways involved in innate immune activation. We observe that monocytes acquire an M1-like profile during early inflammation, and switch to a deactivated M2-like profile during both the resolving and persistent phases. However, during persistent inflammation they partially maintain an M1 profile, although they lose the ability to produce inflammatory cytokines compared to M1 cells. The production of IL-1 family molecules by ELISA reflected the transcriptomic profiles in the distinct phases of the two inflammatory reactions. Based on the results, we hypothesize that persistence of inflammatory stimuli cannot maintain the M1 activated phenotype of incoming monocytes for long, suggesting that the persistent presence of M1 cells and effects in a chronically inflamed tissue is mainly due to activation of newly incoming cells. Moreover, being IL-1 family molecules mainly expressed and secreted by monocytes during the early stages of the inflammatory response (within 4-14 h), and the rate of their production decreasing during the late phase of both resolving and persistent inflammation, we suppose that IL-1 factors are key regulators of the acute defensive innate inflammatory reaction that precedes establishment of longer-term adaptive immunity, and are mainly related to the presence of recently recruited blood monocytes. The well-described role of IL-1 family cytokines and receptors in chronic inflammation is therefore most likely dependent on the continuous influx of blood monocytes into a chronically inflamed site.

## Introduction

Inflammation is the first physiological mechanism of defence against external and internal dangers (e.g., pathogens and foreign materials, or dead cells and Damage Associated Molecular Patterns from tissue injury—sterile inflammation). The inflammatory response progresses through distinctive phases, from initiation to acute phase, followed by resolution and subsequent re-establishment of tissue integrity and function (homeostatic conditions). The acute phase of inflammation may be sufficient to eliminate the dangerous event, but a sustained exposure to triggering agents or an improper reaction against self-molecules could lead to the persistence of the inflammatory reaction (chronic phase) that causes excessive damage to host tissues and potentially degenerates into pathological outcomes (e.g., autoimmune diseases, asthma, atherosclerosis, diabetes, and cancer). For this reason, the inflammatory process must be tightly controlled (1, 2).

Among immune cells involved in the inflammatory reaction, monocytes and macrophages are key players both by directly eliminating foreign agents and as orchestrators of the different phases of the entire inflammatory process (3).

An acute inflammatory reaction is initiated by circulating monocytes that are newly recruited from bloodstream to the site of inflammation within the tissue, and by resident tissue macrophages, which derive from adult monocytes and yolk sac or faetal monocytes [in different percentage depending on the tissue; (4, 5)], present in solid tissues.

Monocytes can enter a tissue in physiological conditions, and differentiate into macrophages in order to replenish the pool of tissue macrophages following homeostatic loss. Conversely, recruited blood monocytes can become inflammatory monocytes or macrophages upon tissue damage (6, 7). Indeed, during inflammation they may transiently persist as monocytes without differentiation and exert a number of functions within the damaged tissue (inflammatory monocytes) (8), or, in turn, they can be reprogrammed and generate tissue macrophages with inflammatory functions (9, 10).

Tissue macrophages can acquire different functional phenotypes upon exposure to surrounding environmental tissue-derived (damage/injury) or cell-derived signals (microorganisms, activated lymphocytes) (11, 12). Although the microenvironmental stimuli and the resulting functional phenotypes are multifaceted, two main macrophage activation profiles have been proposed. Classically activated macrophages (M1) develop in response to microbial challenges and also in response to inflammatory factors like TNF-α and the NK and Th1 cytokine IFN-γ, and mediate resistance against intracellular microbes and tumours (13, 14). Alternative or deactivated macrophages (M2) are either inflammatory macrophages alternative to M1 (i.e., able to inhibit M1 activation and having type 2 inflammatory effects) or tissue-preserving cells that contribute to dampening inflammation, reconstructing and remodelling the tissue and re-establishing homeostasis (15). Thus, during the late phase of the inflammatory response, the transition of the cell reactivity from a cytocidal tissue-damaging mode to a tissue-repairing mode becomes crucial.

Circulating monocytes, monocyte-derived macrophages and tissue macrophages can differ for their origin, morphology, and transcriptomic profile (16–18). However, during the acute phase of inflammatory reaction their functions (i.e., phagocytosis, reactive oxygen/nitrogen species production, antigen presentation, chemokine and cytokine secretion, metalloproteinase release, cell recruitment and so on) are totally overlapping. It is not fully clear whether distinct functional subpopulations are involved in different functions, i.e., if a division of labour exists, although it is evident that tissue macrophages are more involved in phagocytosis and in recruiting other effector cells, while recruited blood monocytes are the main cells responsible of the production of inflammatory mediators (19). In any case, it is evident that monocyte recruitment contributes to local tissue damage during the initial and full phases of the inflammatory reaction, with the secretion of inflammatory and anti-inflammatory cytokines that can lead to disease (20, 21).

In this context, monocyte-derived cytokines become key players of the inflammatory reaction. Actually, it is unknown whether a chronic degeneration of the inflammatory response could be attributed to a “pathological” level of inflammatory cytokines. Currently, the only hallmarks of the chronicity are the persistence of triggering stimuli over time and/or the dysregulation of immune tolerance mechanisms (22). These determine, in the case of excessive response, the risk of chronic inflammation/autoimmunity or, when insufficient, the risk of immunosuppression and increased susceptibility to diseases/infections. Moreover, because of the short half-life of cytokines and their potent activity (which can be detrimental also for normal cells and tissues), under physiological response conditions the majority of these soluble factors are readily neutralised thereby limiting and controlling their bioactivity. Thus, the balance between an effective defensive response and its collateral tissue injury effect depends on the coordinated regulation of cytokine secretion or inactivation, while an imbalance of their regulation may lead to a persistent inflammation. Changes in circulating cytokines are common virtually to all inflammatory conditions, both in resolving and in persistent inflammatory situations, therefore no individual inflammatory cytokine has been yet identified as a specific biomarker associated with pathologic events or with deviation toward a chronic phase. In other words, acute and chronic phases largely overlap in terms of inflammatory cytokine levels, and the only discriminating factor seems to be their production over time. Eventually, the cytokine profile observed in a particular inflammatory condition may depend not only on the degree of stimulation (severity) and how long it persists (chronicity) but also on the individual responsiveness and adaptability of the immune system. This is especially true for the cytokines of the IL-1 family (23).

The IL-1 family includes agonist ligands (IL-1α and IL-1β, IL-18, IL-33, IL-37, IL-36α, β and γ, IL-38); specific antagonist ligands (IL-1Ra and IL-36Ra); receptors (IL-1R1, IL-1R2, IL-1R4, IL-1R5, and IL-1R6) that are both responsible of ligand binding and, in their soluble form, can act as decoy inhibitors; a soluble receptor-like inhibitor of IL-18 (IL-18BP); accessory chains (IL-1R3, IL-1R7) that are responsible of signalling together with the ligand-binding receptors; and orphan receptors (IL-1R8, IL-1R9, and IL-1R10). The cytokines of the IL-1 family are important in regulating the cross-talk between innate and adaptive immunity (24), and play a central role in the activation and regulation of inflammatory responses and in the pathogenesis of wide range of diverse diseases, including cancer and inflammatory and autoimmune disorders (25–27).

Several members of IL-1 family cytokines elicit a strong inflammatory responses that can become harmful if prolonged over time. Thus, their activity is closely monitored at the level of production, protein processing and maturation (28), and also at the level of receptor binding and post-receptor signalling, by naturally occurring inhibitors, e.g., anti-inflammatory cytokines, antagonists and membrane-bound or soluble decoy receptors belonging to the same IL-1 family. An imbalance between agonists and antagonists/soluble receptors can lead to exaggerated inflammatory responses. Notably, some of these cytokines, e.g., IL-18 and IL-33, may play opposite roles (protective or pathogenic) in driving either inflammation or resolution depending on the disease phase (early vs. late) (29).

Therapeutic intervention to down-regulate inflammatory responses is a double-edged sword, considering the importance of inflammation as a physiological defence mechanism, which therefore must have the possibility to develop. In this perspective, current therapies with biologics target specific cytokines, to achieve a decrease of excessive inflammation without disrupting the overall inflammatory mechanism. This is the case of anakinra, a recombinant non-glycosylated form of the natural human IL-1Ra that inhibits the biological effects of IL-1β and that has a significant therapeutic effect on a large number of inflammation-related diseases (30). It remains the fact that these treatments are symptomatic, decreasing inflammation and inflammation-induced tissue damage, but do not address the fundamental issue of how a normal physiological defensive inflammatory response failed to resolve and has become chronic. Thus, it is important to know the kinetics of production of these cytokines during a physiological inflammatory response, and to understand how it diverges from resolution for going toward chronicity/pathology.

In this study, we have profiled the expression and production of IL-1 family cytokines and receptors during the course of a resolving vs. a chronic inflammatory reaction of human monocytes. To this end, we have developed and characterised two *ad hoc in vitro* models able to simulate the resolving and persistent inflammatory reactions carried out by blood monocytes, as they are expected when entering a damaged tissue. We show that IL-1 family molecules are mainly expressed and secreted by monocytes during the early stages of inflammatory response (within 4–14 h), and the rate of their production decreases during the late phase of both resolving and persistent inflammation. This finding shows that IL-1 factors are key regulators of the acute defensive innate inflammatory reaction that precedes establishment of longer-term adaptive immunity, and are mainly related to the presence of recently recruited blood monocytes. The well-described role of IL-1 family cytokines and receptors in chronic inflammation is therefore most likely dependent on the continuous influx of blood monocytes into a chronic inflammatory site.

## Materials and Methods

### Monocyte Isolation and Culture

Human monocytes were obtained from buffy coats of 6 individual adult healthy donors by isolating PBMC on Ficoll-Paque PLUS gradients (GE Healthcare, Bio-Sciences AB, Uppsala, Sweden) and subsequent separation with Monocyte Isolation kit II (Miltenyi Biotec, Bergisch-Gladbach, Germany). Only monocyte preparation with high purity (>98%, evaluated by differential staining on cytospin smears) and viability (trypan blue dye exclusion) were used for the experiments.

Monocytes were cultured at 5 × 10^6^ cells/well in 6-well culture plates (Costar®, Corning Inc., Corning, NY) in 2 ml of RPMI 1640+Glutamax-I Medium (GIBCO®, Life Technologies, Paisley, UK) supplemented with 50 μg/ml Gentamicin (GIBCO®) and 1 or 5% heat-inactivated human AB serum (Sigma-Aldrich Inc., St. Louis, MO, USA) in moist air with 5% CO_2_ or in hypoxic conditions (by using humidified hypoxic chamber with >1% O_2_ balanced with N_2_) (see next paragraph). Monocytes were sequentially exposed to two different sequences of stimuli, in order to simulate the resolving and persistent inflammatory reactions (see next paragraph). *Resolving inflammation*: hrCCL2 (10 ng/ml), hrTNF-α (10 ng/ml), hrIFN-γ (25 ng/ml), hrIL-10 (20 ng/ml), hrTGF-β (10 ng/ml) (all from R&D Systems, Minneapolis, MN), Generalised Modules for Membrane Antigens (GMMA, 30–60 nm LPS-bearing outer membrane particles from *Shigella sonnei* ΔtolR ΔvirG; 30 ng/ml; GMMA kindly provided in 2013 by Novartis Vaccine Institute for Global Health, now part of GSK as GSK Vaccine Institute for Global Health). *Persistent inflammation*: hrCCL2 (10 ng/ml), hrIFN-γ (25 ng/ml), hrM-CSF (0.5 ng/ml), hrGM-CSF (0.5 ng/ml) (all from R&D Systems), Anti-Citrullinated Protein Antibody-containing Immune Complexes (ACPA-IC) (25 μg/ml of antibody plus 6 μg/ml of antigen incubated for 1 h at 37°C; Ab and Ag were isolated from serum of patients affected by Rheumatoid Arthritis); LPS (5 ng/ml; from *E. coli* serotype O55:B5; Sigma-Aldrich Inc.), peptidoglycan (PGN-BS; 1 μg/ml; from *B. subtilis*), Poly(I:C) (500 ng/ml, HMW), CpG (1 μM; ODN 2395) (all from InvivoGen, San Diego, CA, USA), huSurvivin/BIRC5 (1 ng/ml; BioVision Inc., Milpitas, CA, USA).

Cells were washed and fresh medium with different stimuli added at 2, 14, and 24 h for the resolving model and at 2, 7, 24, and 72 h for the persistent model. In the resolving model, TNF-α at 3 h and IFN-γ at 7 h were added without washing. Viability at 48 h and 96 h always exceeded 80 and 50%, respectively. Fresh monocytes were taken as time 0. Cells were harvested in 700 μl of Qiazol (Qiagen, Hilden, Germany) at 0, 2, 4, 14, 24, and 48 h for the resolving model, and at 0, 2, 4, 14, 24, 72, and 96 h for the persistent model. Supernatants were collected at the same time points, except for time 0.

### The *in vitro* Monocyte-Based Models of Inflammation

**Figure 1:**
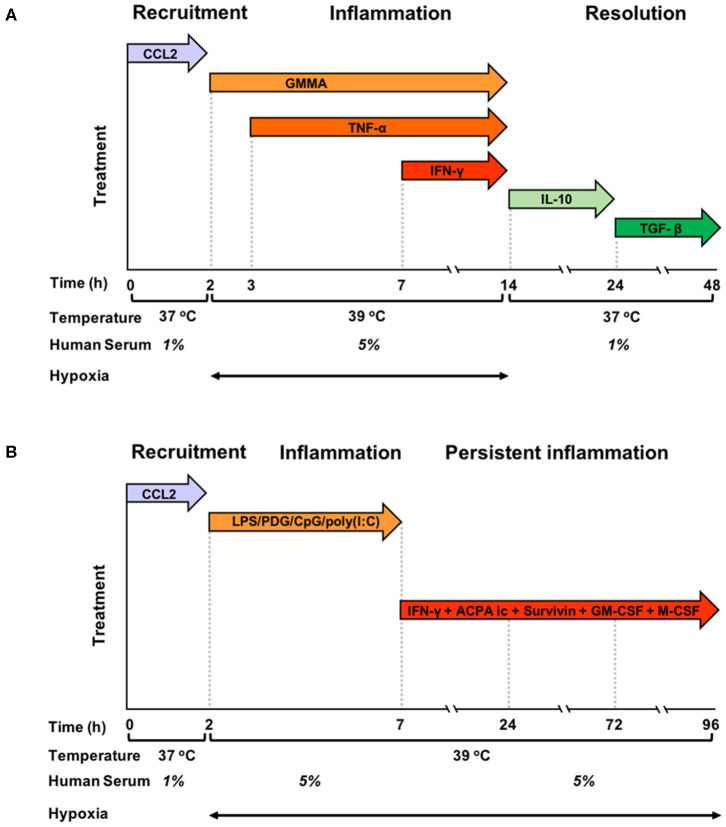
Graphic representation of the kinetic development of resolving and persistent inflammation in the human monocyte-based *in vitro* models. **(A)** Freshly isolated human blood monocytes were first exposed to the chemokine CCL2 for 2 h at 37°C with 1% serum in normoxic conditions, then, after washing, to GMMA (from 2 h), TNF-α (from 3 h, without washing), and IFN-γ (from 7 h, without washing) at 39°C with 5% serum in hypoxic conditions. At 14 h, the inflammatory stimuli were washed off, temperature and serum concentration brought back to 37°C and 1%, respectively, and fresh medium containing IL-10 added. At 24 h, IL-10 was washed off and monocytes were exposed to TGF-β until the end of the experiment. **(B)** Freshly isolated human blood monocytes were first exposed to the chemokine CCL2 for 2 h at 37°C with 1% serum in normoxic conditions, then, after washing to LPS, PDG, poly(I:C), and CpG (from 2 to 7 h) at 39°C with 5% serum in hypoxic conditions. At 7 h, the inflammatory stimuli were washed off, and fresh medium containing ACPA complexes, GM-CSF, M-CSF, Survivin, and IFN-γ was added. The temperature was kept to 39°C, serum at 5% and oxygen tension at hypoxic levels until the end of the experiment. Cells were harvested at 0, 2, 4, 14, 24, and 48 h for the resolving model, and at 0, 2, 4, 14, 24, 72, and 96 h in the persistent model. Supernatants were collected at the same time points, except for time 0.

Blood monocytes were exposed to a sequence of different stimuli and kept in different culture conditions, as described in the preceding paragraph, which were designed to mimic the evolving tissue microenvironment during resolving or persistent inflammatory reactions (Figures 1A,B). In the resolving model (Figure 1A), monocytes were initially exposed to CCL2 at 37°C with 1% human serum, in order to reproduce the recruitment to the site of infection, then to bacterial vesicles (GMMA) and, sequentially, to TNF-α and IFN-γ at 39°C in hypoxic condition and 5% serum, to simulate the encounter with pathogens and the development of an inflammatory microenvironment (tissue reaction in terms of heat production, oedema formation and influx of NK and Th1 cells). At 14 h, culture conditions were changed (37°C, 1% serum, normoxia, and medium containing IL-10 first and subsequently TGF-β) to reproduce activation of anti-inflammatory mechanisms and macrophage deactivation during the resolution phase of the reaction. In the persistent model (Figure 1B), monocytes were initially exposed to CCL2 at 37°C with 1% human serum (the same as in the resolving model), then to a mixture of TLR-activating microbial molecules [LPS, PGN, CpG, poly(I:C)], to mimic a generalised infection/inflammatory challenge, at 39°C in hypoxic conditions and 5% serum. At 7 h, monocytes were further stimulated with immune complexes, growth factors, IFN-γ and Survivin to reproduce a persistent inflammatory condition without resolution, similar to that observed in the microenvironment of Rheumatoid Arthritis joints. Culture conditions, i.e., high temperature, high serum and hypoxia, were kept constant until 96 h.

### RNA Isolation and RNASeq

Total RNA was extracted from monocytes of 6 individual donors (3 for the “resolving model”: 0, 4–48 h; and 3 for the “persistent model”: 0, 4–96 h), using Qiagen miRNeasy kit (Qiagen), quantified spectrophotometrically (ND-1000; NanoDrop Technologies, Wilmington, DE), and checked for integrity by microcapillary electrophoresis (Agilent 2100 Bioanalyzer; Agilent Technologies, Palo Alto, CA).

RNA-seq was performed at BGI Genomics (Shenzhen, China). Libraries were prepared using TruSeq RNA Sample Prep Kit v2 (Illumina, Inc., San Diego, CA, USA) and sequenced with an Illumina HiSeq2000 sequencer (Illumina), using paired-end reads of length 90 bp. HiSeq Control Software, Real-Time analysis and Off-Line Basecaller (all from Illumina) were used for base-calling and adapter trimming. RNA-seq data have been deposited in the ArrayExpress database (31) at EMBL-EBI (www.ebi.ac.uk/arrayexpress) under accession number E-MTAB-8226.

Raw reads were aligned to RefSeq (hg19) using SOAPaligner/SOAP2. Missing values in count matrix (genes-by-samples) were set equal to the average of the other two replicas, while if only one replica was available the other two were set to zero. Raw counts were normalised using the TMM method (trimmed mean of M values) (32) available in R package edgeR (33) and log-cpm (count-per-milion) values were obtained using the “voom” function available in R package limma (34). Only genes with cpm >1 in at least 4 samples were kept. Normalised gene expression values were available for a total of 13,258 genes in 36 samples.

### Analysis of Gene Expression Data

Principal component analysis was carried out using the R package “pcaMethods” (35). Gene expression variations were assessed using moderated t-statistics and moderated F-statistics (36) implemented in limma (34). Nominal *P*-values were corrected using the Benjamini-Hochberg (BH) procedure implemented in the R function “p.adjust.” Genes were ranked by the score s =–*log*_10_(*q*)^*^log_2_(FC) (37) where *q* is the BH adjusted *p*-value and FC is the fold change; therefore, the higher the absolute value of *s* the more likely the gene is differentially expressed.

### Pathway Analysis

NCBI Biosystems (38) and mSig databases (39) were used as sources of annotated gene sets referring to pathways and molecular signatures. Only gene sets with at least 10 genes and not more than 500 genes were considered. Enrichment was assessed using both gene set enrichment analysis (GSEA) (39) and hypergeometric test (over-representation analysis, ORA). For each gene set, the hypergeometric test was applied considering the top 750 genes ordered by decreasing value of |s*|*. Gene set rankings determined by GSEA and ORA were integrated by means of the rank product, i.e., the geometric mean of pathway ranks in GSEA and ORA.

### Cytokine Measurements

The IL-1 family cytokines (IL-1α, IL-1β, IL-18, IL-33, IL-36β, IL-36γ), receptors and accessory proteins (sIL-1R1, sIL-1R2, sIL-1R3), IL-1 receptor antagonist (IL-1Ra) and the IL-18 natural inhibitor (IL-18BP) were measured using a multiplex assay technology and software custom-developed by Quansys Biosciences, Inc. (Logan, UT, USA), and validated in-house for sensitivity, specificity, robustness and reliability of measurement. IL-36β, IL-36γ, and IL-36 receptor antagonist (IL-36Ra) were measured by ELISA (R&D Systems, Minneapolis, MN), according to manufacturer's instruction. Each sample was assayed in duplicate. The rate of cytokine production was assessed as cytokine production relative to the number of living cells at each given period of time (pg/10^6^ cells/h).

### Assessment of Free and Active IL-1β and IL-18

The calculation of free IL-18 and IL-1β (i.e., the fraction of cytokine not bound to its soluble inhibitor) in cell culture supernatants has been evaluated by applying the law of mass action, as previously described (40, 41) and shown below. For IL-18, the calculation considers the measure of the soluble inhibitor IL-18BP. For IL-1β, the calculation considers sIL-1R2 as main soluble inhibitor in culture supernatants, and disregards the levels of sIL-1R1 and IL-1α, which are low. Thus, the major soluble ligand of IL-1β is sIL-1R2, a molecule that has good affinity for IL-1β (calculated as 2.7 nM) but a significantly lower affinity for IL-1Ra (25 μM) (42). The law of mass action was therefore adapted to account for the free ligand concentration ([LF], see below), according to Clark's theory (i.e., one ligand, one receptor, specific binding):

$$[\text{LF}]=\frac{-[\text{RT}]+[\text{LT}]-\text{Kd }+\sqrt{{\left(\left[\text{RT}\right]-\left[\text{LT}\right]\text{​}+\text{​Kd}\right)}^{\text{2}}\text{+4}\left[\text{LT}\right]\;\times \text{ Kd}}}{\text{2}}$$

where:

RT: pM concentration of sIL-1R2 (MW 47 kDa) or of IL-18BP (MW 40 kDa);

LT: pM concentration of IL-1β (MW 17 kDa) or of IL-18 (MW 18 kDa);

Kd: dissociation constant IL-1β/sIL-1R2 (2700 pM; 42, 43) or IL-18/IL-18BP (400 pM; 44).

An additional calculation was done by considering that one part of sIL-1R2 is engaged with sIL-1R3 (MW 47 kDa) in forming with sIL-1R2 higher affinity complexes for IL-1β [5.6 pM; (43)], this being dependent on the concentration of available sIL-1R3. Free IL-1β was thus the concentration of IL-1β not engaged in low affinity soluble complexes with sIL-1R2 and in high affinity soluble complexes with sIL1R2 and sIL-1R3. Eventually, active IL-1β was calculated as the ratio between free IL-1β and IL-1Ra, multiplied by 1000:

$$\begin{array}{l} Active\;IL-1\beta =\left(free\;IL-1\beta /IL-1Ra\right)\;\times \;1000. \end{array}$$

### Statistical Analysis of Protein Production

The ELISA results are shown as single values. Differences between time points were analysed using one-way ANOVA and Tukey's multiple comparisons test using GraphPad Prism 7.0. A *P-*value < 0.05 was considered statistically significant.

### Ethics Statement

The ongoing study on human monocyte activation was approved by the Ethical Committee of the University of Pisa S. Chiara Hospital (prot. AOUP 33998 of September 29, 2006). Human blood used in this work was taken from volunteers after informed consent. Donors were anonymous to the researchers.

## Results

### Macrophage Polarisation: Inflammatory vs. Deactivated Macrophages

In a previous study, we demonstrated that macrophages with an M1 inflammatory signature develop into M2 (deactivated) during the resolution phase of the *in vitro* model of resolving inflammation (44). To evaluate the shift from M1 to M2, we identified a list of genes differentially expressed in fresh monocytes, M1 and M2 macrophages by meta-analysis. The statistical comparison returned that monocyte-to-M1 differentiation was associated with modulation of 98 specific genes (up-regulated in fresh monocytes and down-regulated in M1 cells, or *vice versa*), while monocyte-to-M2 differentiation resulted in the modulation of 107 specific genes (up-regulated in fresh monocytes and down-regulated in the M2 cells, or *vice versa*) (44) (Supplementary Figures 1, 2, and Supplementary Tables 1, 2). We have used these two lists to cluster samples of our two kinetic models of resolving and persistent inflammation and compare their profiles. The acquisition of an M1 profile is evident in the two models starting from the early inflammation phases (Supplementary Figure 1). In the resolving model, the M1 profile is evident up to 14 h (the time point in which the resolution begins), while during resolution (from 24 to 48) it tends to return to a baseline monocyte-like expression profile (in line with previous results; (44). On the other hand, in the persistent model the M1 gene expression profile is largely maintained up to 24 h, and for several of these genes even until 96 h. Regarding the M2 profile (Supplementary Figure 2), the signature is similar in the two models, showing the transition with time from the baseline monocyte expression profile at time zero to the M2 profile, with up-regulation of poorly expressed genes starting earlier and down-regulation of highly expressed genes more evident starting at 14 h. There are no obvious differences in the shift toward M2 polarisation between the resolving and persistent inflammatory reactions. From these results, we can infer that monocytes acquire an M1-like profile during early inflammation and switch to a deactivated M2-like profile during the resolution phase, while in a persistent inflammatory reaction they partially maintain an M1 profile and at the same time acquire an M2 profile in the late phases of persistent inflammation.

### PCA Confirms Differences Between the Gene Expression Profiles in the Two *in vitro* Models of Resolving and Persistent Inflammation

**Figure 2:**
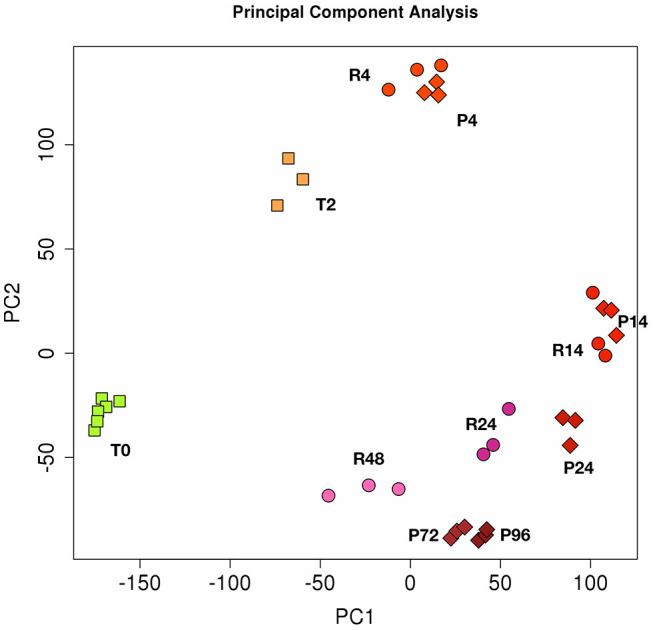
Principal component analysis (PCA) of gene expression in resolving and persistent inflammatory models. Each dot represents a sample. Six samples (monocytes from 6 different donors) were analysed at time 0 (T0) and three after 2 h with CCL2 (T2). Three samples were examined for each time point of the two models after sequential stimulations as described in the Figures 1A,B (3 donors for the resolving model, R; and 3 donors for the persistent model, P). Numbers represent the time points: 4, 14, 24, and 48 h for resolving inflammation; 4, 14, 24, 72, and 96 h for persistent inflammation. The samples cluster in different areas from left to right, based on the time points of stimulation, which correspond to different phases of inflammatory reactions.

The Figure 2 shows the PCA (Principal Component Analysis) of gene expression in the two *in vitro* models of inflammation. Biological replicates (gene expression in individual donors) always cluster together, as well as samples at different time points, proving that gene expression profiles change in the same way in all donors during the different phases of an inflammatory reaction. While the resolving and persistent models are very similar at 4 and 14 h in terms of gene expression, they clearly diverge from 14 h on, i.e., when resolution starts in the resolving model and inflammation is maintained in the persistent model.

### Distinctive Gene Signatures Are Observed During the Resolving and Persistent Inflammation

Transcriptomic analysis was performed on monocytes from each donor at four different phases of activation in the resolving model (4, 14, 24, 48 h) and at six phases in the persistent model (2, 4, 14, 24, 72, 96 h), in comparison to control fresh monocytes (time 0). The time points are: early inflammation (2–4 h), late inflammation (14 h) (both corresponding to M1 polarisation) in both models; early and late resolution (24 and 48 h) (different stages of deactivated M2 polarisation) in the resolving model; late and persistent inflammation (72 and 96 h) in the persistent model.

**Figure 3:**
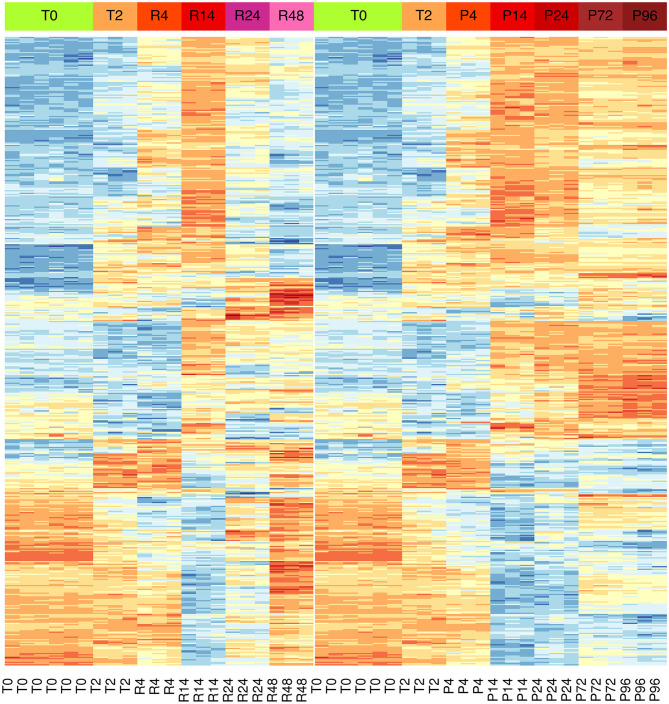
Differential gene expression during the inflammation phases of the resolving and persistent inflammatory models. Heat-maps of the 500 genes with the most significant expression differences (limma moderated F-statistics, FDR < 0.05) between the two models at any time point (Supplementary Table 3). Left: resolving inflammation model; right: persistent inflammation model. The gene clusters show that the two models are very similar from 0 to 14 h, but they begin to diverge from 14 to 24 h. Colors represent gene expression (gene-wise z-score); blue: low; red: high.

Results showed significant changes in gene expression during the different stages of the inflammatory reactions, and the associated monocyte-to-macrophage differentiation. The list of the 500 genes with the most significant differences between the two models (FDR << 0.05) is shown in the Supplementary Table 3. As in the case of PCA analysis, the heat-map of these genes underlines a divergence from 14 to 24 h (Figure 3). It is evident that a large part of genes that are up-regulated at 14 h and down-regulated at 24–48 h in the resolving model remain up-regulated until to 96 h in the persistent model, while genes down-regulated at 14–24 h and up-regulated again at 48 h in the resolving model remain down-regulated in the persistent model. Also, in the persistent model most of the genes are expressed at opposite levels respect to monocytes, while these largely return to baseline in the resolving model.

### Pathway Analysis Reveals Innate Immune Activation in the Resolving and Persistent Inflammatory Responses

**Figure 4:**
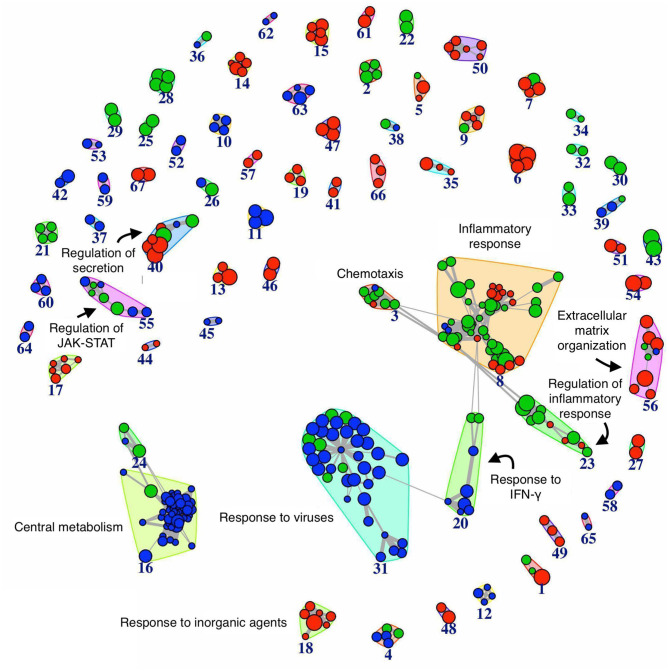
Network of biological pathways enriched in differentially expressed genes between resolving and persistent inflammation. Network of pathways significantly enriched (see Materials and Methods) in genes differentially expressed between the two models at 24 h, and between resolving inflammation at 48 h (R48) and persistent inflammation at 96 h (P96). The bigger the circle, the higher the number of genes in the pathway. Circle colors reflect pathway occurrence among the top 500 pathways at 24 h only (blue), between P96 vs. R48 (red), or both (green). Links are reported only between any pathway pair (X, Y) with overlap coefficient $O\left(X,Y\right)=\frac{\left|X\cap Y\right|}{\min\left(\left|X|,|Y\right|\right)}\geq 0.7$. See Supplementary Tables 4, 5.

We performed the pathway analysis for those time points where we observed a divergence between the resolving and persistent inflammation, that is at 24 h (when the inflammation resolves in the resolving model while persisting in the persistent model) and at 48 vs. 96 h (the latest time points in the two models) (see Figure 2). In order to find statistically significant associations between expression profiles of distinct groups and gene signatures typical of several biological pathways or cellular processes, pathway analysis was performed on gene groups that differ at 24 h and at 48 vs. 96 h (Supplementary Tables 4, 5). The enrichment map (45) depicted in the Figure 4 summarises the similarity (in terms of gene content) between the top significant biological pathways enriched in differentially expressed genes at the considered time points (see section Materials and Methods). As expected, at 24 h the genes involved in the central metabolism are differentially expressed between the two models, underlining the extent by which a metabolic remodelling (metabolism and energy expenditure) matches with different functions acquired by monocytes in different phases of the inflammatory responses. At 24 h we can also observe a significant difference in pathways involved in the response to viruses and IFN-γ signalling, compatible with the stimulation with viral TLR agonists and the continuous presence of IFN-γ in the persistence inflammation model while absent in the resolving model. Among the most different pathways between persistent and resolving inflammation both at 24 h and at the final time points (48 and 96 h), we find several pathways involved in chemotaxis, inflammatory response and its regulation, again underlining the notion that inflammatory activities are distinctly regulated in persistent vs. resolving reactions. The regulation of JAK-STAT signalling (a rapid membrane-to-nucleus signalling module for a wide array of cytokines and growth factors) seems to be differentially modulated starting at 24. We have further examined the expression of a number of genes related to the JAK/STAT pathway (see list in https://www.ncbi.nlm.nih.gov/biosystems/?term=83077) (Supplementary Figure 3). We can observe that most of them are upregulated between 4 and 14 h in both models, and many remain upregulated in the persistent model. Among them, we observe that the four JAKs (JAK1, JAK2, JAK3, and TYK2) and the seven STAT family members (STAT1, STAT2, STAT3, STAT4, STAT5a, STAT5b, STAT6) are differentially expressed at different time points in the two models. In particular, considering the time frame 24–48 h for the resolving model and 24–96 h for the persistent model, we observe that JAK1/2-STAT1/3 [important for signalling by type I IFNs and involved in M1 polarisation; (46)] are down-regulated in the resolving model and up-regulated in the persistent model, while for the axis JAK1/2/3-STAT6 [important for M2 polarisation; (46)] we observed an up-regulation of JAK2/3 and a strong down-regulation of STAT6 in the persistent model. The profiles of expression of these genes, as well as the others belonging to the pathway, mirrors the functional data showing the development of an M2 phenotype in monocytes in the resolving model and the maintenance of the M1 phenotype in the persistent model. The majority of genes involved in the regulation of secretion appear to be differentially expressed during the final phases of the two inflammatory reactions (48 and 96 h). During these final phases, it is also evident a differential expression of genes involved in the extracellular matrix organization, indicating that the involvement of macrophages in this biological process is very different in resolving vs. chronic inflammation. Likewise, a protective innate immune response against a harmful overdosing of inorganic agents/metals seems to be activated during the late phases of the inflammatory reactions.

### IL-1 Family Members Emerge Among the Genes Differentially Expressed at Different Time Points in Resolving and Persistent Inflammation

**Figure 5:**
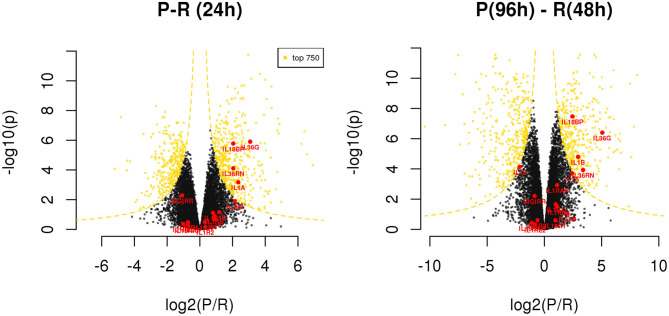
Differentially expressed genes between resolving and persistent models. For each comparison, genes are reported on considering BH adjusted *P*-value (y axis) and log_2_ fold change (x axis); the yellow dotted lines depict the hyperboles that separate the top 750 genes (yellow points) ranked by *s* score (see Materials and Methods), i.e., the product between the quantities reported on the two axes. See Supplementary Table 6.

Exploring the differences between resolving and persistent inflammation by comparing the critical time points 24 h (P vs. R) and 96 h (P) vs. 48 h (R), we performed a statistical analysis enabling quick visual identification of genes displaying large statistically significant magnitude changes (see Supplementary Table 6). As shown in the Volcano Plots in the Figure 5, among these genes we found several members of IL-1 family. At 24 h, only the cytokine genes *IL1A* and *IL36G*, and the inhibitor genes *IL18BP* and *IL36RN* are significantly up-regulated in the model of persistent inflammation respect to resolving inflammation, with *IL18BP* and *IL36G* being the most significant. Comparing the final time points of the two inflammation models (96 h for the persistent inflammation model with 48 h for the resolving inflammation model), we found up-regulation of the same genes *IL36G, IL18BP*, and *IL36RN* in persistent inflammation, and the up-regulation of *IL1B* replacing *IL1A*.

### Modulation of IL-1 Family Gene Expression During Resolving and Persistent Inflammation

**Figure 6:**
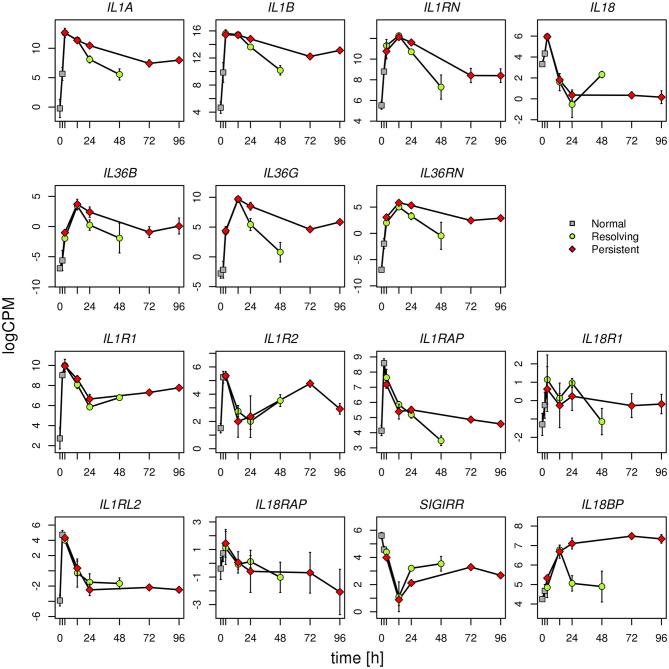
Gene expression profiles of IL-1 family cytokines and receptors. Expression levels of the genes of IL-1 family during the resolving and persistent inflammatory reactions. The mean expression values from three different donors for each model are reported. logCPM: log count per million. Bars indicate standard deviation across biological replicates. Statistically significant differences are as follows (R = resolving; P = persistent): *IL1A* R24 vs. P24 *P* < 0.001; R48 vs. P96 *P* < 0.0001 *IL1B* R48 vs. P96 *P* < 0.0001 *IL18* R48 vs. P96 *P* < 0.0001 *IL36B* R24 vs. P24 *P* < 0.05 *IL36G* R24 vs. P24 *P* < 0.0001; R48 vs. P96 *P* < 0.0001 *IL36RN* R24 vs. P24 *P* < 0.0001; R48 *v*s. P96 *P* < 0.001 *IL1RAP* R48 vs. P96 *P* < 0.05 *SIGIRR* R24 vs. P24 *P* < 0.05; R48 vs. P96 *P* < 0.05 *IL18BP* R24 vs. P24 *P* < 0.0001; R48 vs. P96 *P* < 0.0001 All other differences have a *P* > 0.05.

Given the evidence from Volcano Plot analysis and the central role of IL-1 family members (cytokines, inhibitory proteins and receptors) in inducing and regulating inflammation, we have examined their expression more in detail. RNASeq data show that expression of the cytokine genes *IL33, IL36A, IL37*, and *IL38*, and the receptor genes *IL1RL1, IL1RAPL1*, and *IL1RAPL2* is negligible and not different in monocytes during the entire course of the two inflammatory reactions (Supplementary Table 6). All the other genes of IL-1 family are expressed and show several statistically significant expression changes over time and between the two inflammatory reactions. As shown in the Figure 6, and in agreement with the PCA (Figure 2), from 0 to 14 h the gene expression profile is practically identical for all receptors and cytokines in the two models. In general, in both models the cytokine genes (*IL1A, IL1B, IL1RN, IL36B, IL36G, IL36RN*, and also *IL18BP*) are significantly up-regulated up to 14 h (except IL-18 that is up-regulated at 4 h and decreased at 14 h), while the receptors and accessory protein genes (*IL1R1, IL1R2, IL1RAP, IL18R1, IL1RL2, IL18RAP)* are up-regulated at 4 h and down-regulated from 4 to 14 h. Again, similar to PCA, the genes that significantly differed between persistent and resolving inflammation from 14 to 24 h are the cytokine genes *IL1A, IL36B*, and *IL36G*, and the receptor antagonist gene *IL36RN*, the inhibitory receptor gene *SIGIRR*, and the gene for the natural IL-18 inhibitor *IL18BP*. Moreover, while *IL1R1, IL1R2, IL1RL2*, and *IL18RAP* genes maintain the same profile for the entire course of both resolving and persistent inflammation (increasing during the initial phase and decreasing thereafter), all other genes remained up-regulated at the late time points of the persistent inflammatory reaction respect to the resolving reaction, although not increasing respect to 24 h. An exception is the *IL18BP* gene that remained very highly expressed during the entire course of persistent inflammation, likely because of the presence of its major inducer IFN-γ. As an exception, after an increase at 4 h, *IL18* expression decreased below the basal level at 14 and 24 h in both models and returned up to basal levels at 48 h only in the resolving inflammatory reaction, while remaining very low in the persistent inflammatory response. A distinct profile is evident for the anti-inflammatory receptor gene *SIGIRR*, whose expression decreased below the basal level during the initial phases of both resolving and persistent inflammatory reactions (4 and 14 h), and tended to increase again in both systems although never reaching the initial expression levels. In agreement with the Volcano Plot analysis, we observe that expression of *IL18BP, IL36G, IL1A*, and *IL36RN* is increased at 24 h in persistent inflammation respect to resolving inflammation. Likewise, at the last time point of the persistent inflammatory reaction (96 h) the *IL18BP, IL36G, IL36RN, IL1A*, and *IL1B* genes are more expressed respect to the end of resolving inflammation (48 h). Comparing these two time points, only *SIGIRR* and *IL18* remain higher in the resolving model.

### Modulation of IL-1 Family Protein Production During Resolving and Persistent Inflammation

**Figure 7:**
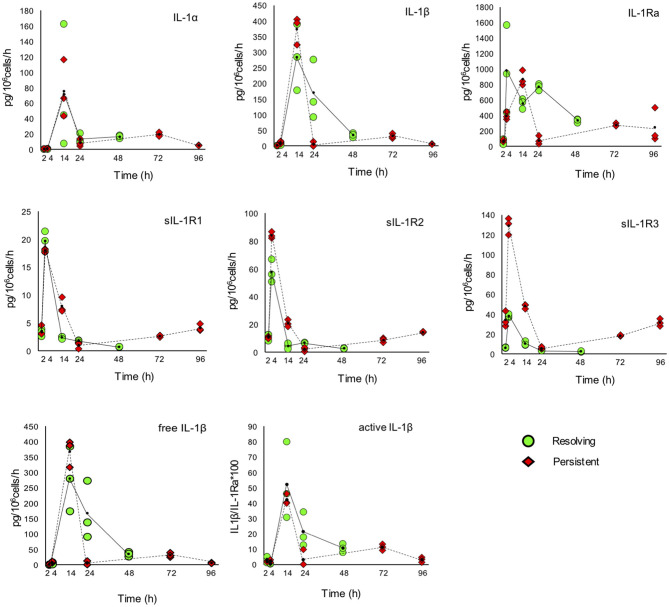
Production of IL-1 family cytokines and receptors during resolving and persistent *in vitro* inflammatory reactions. Production of IL-1 family cytokines and soluble receptors during the resolving (green circles, continuous line) and persistent (red triangles, dashed line) *in vitro* inflammatory reactions. Production of soluble proteins released in the supernatant is reported in terms of rate of production, i.e., the amount of protein produced per million cells per hour. The individual values of three different donors are reported, with a small black dot representing the mean value. Statistical significance was calculated with one-way ANOVA followed by Tukey's multiple comparisons test for significant differences between two consecutive experimental time points within a model and between the two models. A *P*-value < 0.05 was considered statistically significant. The full statistical evaluation is reported in the Supplementary Tables 7, 8.

**Figure 8:**
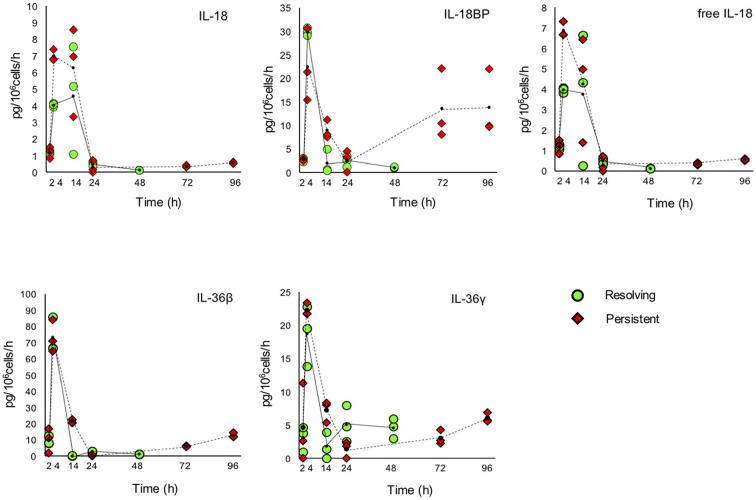
Production of IL-1 family cytokines and receptors during resolving and persistent *in vitro* inflammatory reactions. Production of IL-1 family cytokines and soluble receptors during the resolving (green circles, continuous line) and persistent (red triangles, dashed line) *in vitro* inflammatory reactions. Production of soluble proteins released in the supernatant is reported in terms of rate of production, i.e., the amount of protein produced per million cells per hour. The individual values of three different donors are reported, with a small black dot representing the mean value. Statistical significance was calculated with one-way ANOVA followed by Tukey's multiple comparisons test for significant differences between two consecutive experimental time points within a model and between the two models. A *P*-value < 0.05 was considered statistically significant. The full statistical evaluation is reported in the Supplementary Tables 7, 9.

The kinetic profile of protein production was assessed by ELISA for ten IL-1 family members, i.e., five cytokines of the family (IL-1α, IL-1β, IL-18, IL-36β, IL-36γ, which are encoded by the genes *IL1A, IL1B, IL18, IL36B*, and *IL36G*); three natural inhibitors IL-1Ra (the IL-1 receptor antagonist encoded by *IL1RN*), sIL-1R2 (the soluble form of the IL-1 receptor type 2, encoded by *IL1R2*), the IL-18 inhibitor IL-18BP (encoded by the *IL18BP* gene); two soluble receptors (sIL-1R1 and sIL-1R3, encoded by *IL1R1* and *IL1RAP*, respectively) (Figures 7, 8). We did not measure the soluble form of IL-1R5, IL-1R6, and IL-1R7 (encoded by the *IL18R1, IL1RL2*, and *IL18RAP* gene) due to unavailability of suitable detection reagents or the lack of sensitivity of the currently available detection assays. A soluble form of IL-1R8 (encoded by the *SIGIRR* gene) has never been described. In agreement with expression results, the production of soluble IL-33 was essentially undetectable, while IL-36Ra (the IL-36 receptor antagonist encoded by *IL36RN*) is not produced/released despite detectable gene expression (data not shown). For IL-1α and IL-33, which are expected to be also present within cells and act as alarmins, we have measured their cell-associated levels. As expected, IL-33 was undetectable. On the other hand, cell-associated IL-1α was detectable at significant levels. The kinetics of IL-1α level changes, as measured in the model of the resolving inflammation, shows that intracellular IL-1α increases at earlier times after initiation of inflammation, compared to the extracellular levels (Supplementary Figure 4), and decreases thereafter. There is a clear overlapping between gene expression (Figure 6) and the profile of intracellular IL-1α, while the secreted cytokine levels clearly depend on additional control mechanisms. The production of other factors, which have undetectable expression level, was not assessed (IL-36α, IL-37, IL-38, soluble forms of IL-1R4, IL-1R9, and IL-1R10).

It should be noted that the soluble protein production is presented in the Figures 7, 8 as production per million viable cells per hour, so what we observe is the actual production in the time range considered, rather than the cumulative amount of cytokines present at a given time. The statistical analysis for the comparison of each time range of the two models and for comparison of differences between single time points within and between models is reported in the Supplementary Tables 7–9. Similar to gene expression results, all the IL-1 family members measured are highly produced during the first hours of the inflammatory reaction. The highest rate of production is from 2 to 4 h or from 4 to 14 h for practically all cytokines and soluble receptors, and for the IL-18 inhibitor IL-18BP, in both models. The same trend was observed for IL-1α and IL-1Ra, although but not always statistically significant. While the rate of cytokine and soluble receptor production generally returns to basal levels at the end of the acute inflammatory reaction (24 h), some of them, in particular soluble receptors and inhibitors, are still present at low but significant levels at the later time points of the persistent inflammatory reaction (72 and 96 h). The increased levels of IL-18BP are most likely due to the presence of IFN-γ in culture. Significant differences between resolving and persistent inflammation are evident at 24 h for IL-1β and IL-1Ra, which are produced more abundantly in resolving inflammation, and at 14 h for all soluble receptors, produced at higher rate in persistent inflammation. The production rate of sIL-1R3 is distinctively different between the resolving and persistent inflammation, being consistently higher during the persistent inflammatory reaction.

We have additionally calculated the levels of free active IL-1β and IL-18, i.e., the levels of the two cytokines that are still free/active after interaction with their soluble inhibitors. Calculations are based on the law of mass action (see Materials and Methods) and consider the interaction of IL-1β with its major soluble inhibitor sIL-1R2 plus the contribution of sIL-1R3 in forming higher affinity inhibiting complexes and the ratio with its receptor antagonist IL-1Ra, and the interaction of IL-18 with its soluble inhibitor IL-18BP. The profiles of free and active IL-1β and IL-18 match quite closely those of the total cytokines and confirm the presence of the active cytokines in the early inflammatory phases and their shut-off at later times (Figures 7, 8).

**Figure 9:**
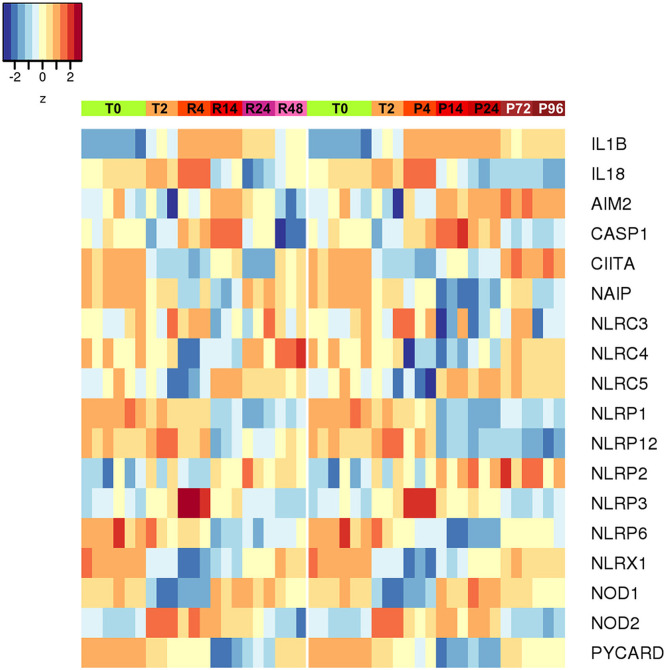
Differentially expressed inflammasome and NLR genes during resolving and persistent *in vitro* inflammatory reactions. Heat-maps represent fold-expression levels of the inflammasome-related and NLR genes in the *in vitro* models of resolving (left) and persistent inflammation (right). Time points are indicated as described for Figure 2.

An additional evaluation was performed on the expression of inflammasome-related genes, in the attempt of better defining the role of the inflammasome-dependent cytokines IL-1β and IL-18. As shown in the Figure 9, the expression of the genes encoding caspase-1 (the enzyme responsible for IL-1β and IL-18 maturation) and NLRP3 (the NLR in the major caspase-1 activating inflammasome) was upregulated during inflammation and decreased thereafter (both in resolving and persistent inflammation). Many of the NLR genes do not show notable expression differences between resolving vs. persistent inflammation, except for some. NLRP12 (which is not related to inflammasomes) is down-regulated in persistent inflammation while expressed at normal levels in resolving inflammation, in agreement with its putative role as regulator of inflammatory activation (47). Most interestingly, the expression of the NLRC4 and NAIP genes is downregulated during inflammation and upregulated during resolution, but only partially at later times in the persistent model. Conversely, the AIM2 gene is upregulated during inflammation and then strongly downregulated during resolution, while further upregulated in persistent inflammation. This expression pattern suggests some important considerations. First, the NLRP3 inflammasome is likely very important during the initial/acute phases of inflammation. In a resolving inflammation, the IL-1β/IL-18 producing inflammasome appears to shift from NLRP3 to NAIP/NLRC4, leading to the hypothesis that cells that have resolved an acute bacterial-induced inflammation become more prepared to react to intracellular bacteria [being the NAIP/NLRC4 inflammasome reactive to those; (48)]. On the other hand, during a persistent inflammation the is an apparent shift from NLRP3 to AIM2, an inflammasome reactive to altered or mislocalised DNA molecules (49), a finding that suggests that, during persistent inflammation, cells may be more prone to react to self-DNA, thereby driving autoimmune reactions.

In order to examine the contribution of cell differentiation (monocytes to macrophages) to the changes in the production of IL-1 family cytokines during time in culture, we assessed the reactivity to inflammatory stimulation of monocytes kept in culture for different time lengths. Monocytes were first cultured for 48 or 96 h (therefore differentiating into macrophages) and then stimulated with LPS. We have measured the production of three IL-1 family proteins (IL-1β, IL-1Ra, and sIL-1R2) and one unrelated inflammatory cytokine, IL-6. As shown in the Supplementary Figure 5, IL-6 is produced at significant levels by LPS-stimulated monocytes previously cultured for 48 h (2 days), and this production increased in cells pre-cultured for 96 h (4 days), showing that these cells are still very reactive. Regarding IL-1 family proteins, we observed different profiles: IL-1β was produced at the same level by 2-day and 4-day old cells; IL-1Ra was highly produced only by 4-day old cells; sIL-1R2 production did not depend on LPS stimulation and was only slightly reduced in 4-day vs. 2-day old cells. Overall, we can say that upon *in vitro* culture monocytes/macrophages can still produce inflammation-related factors in response to stimulation, with some factors actually being produced at higher levels by older cells (i.e., more differentiated into macrophages) and others being produced at stable levels.

## Discussion

In this work, we designed and set up two *in vitro* models of resolving and persistent inflammation based on human primary monocytes, aimed at describing and reproducing the kinetics of the monocyte-dependent inflammatory response from initiation and development until its resolution or persistence. The use of peripheral blood monocytes allowed us to study the reactivity of the very same cells that are recruited *in vivo* in a tissue upon damage or infection. Despite the inter-individual variability, the gene expression and protein production profiles of the donors appeared to be similar, suggesting that the monocyte response is highly reproducible and robust. This study is exclusively focussed on the reactivity of newly recruited monocytes during an inflammation reaction, and the two *in vitro* models do not include resident tissue macrophages nor other cell types present in the tissue *in vivo*. Even with this limitation, the advantage of the two models are the simplicity and the kinetic description of monocyte reactivity during the entire course of resolving and persistent inflammatory responses.

From the transcriptomic analysis, we made two major observations: (1) distinct clusters of genes characterize the different phases of the two inflammatory reactions; (2) these clusters are identical in both models until 14 h and diverge at 24 h when the inflammation resolves in one model while persisting in the other one. The latter genes may be those mainly involved in the chronicisation of inflammation over time. In the pathway analysis we observed that these genes are exclusively associated with pathways involved in innate immune activation, e.g., those associated with the inflammatory response and its regulation. We also found a differential expression of pathways involved in the central metabolism, mostly those related to glycolysis. Glycolysis is closely related to an inflammatory functional phenotype in macrophages (50), and it is known that classical inflammatory M1 macrophages heavily dependent on glycolysis to produce ATP (51).

As previously shown for the resolving model (44), we observe that monocytes first polarise into M1 and then switch to M2 upon microenvironmental changes. This occurs in both models although more clearly in the resolving one, where the resolution phase brings about a clear shut-off of the M1 activation profile. In the persistent model this switch is less clear, with cells partly maintaining an M1 profile but nevertheless acquiring an M2 phenotype. Thus, we can say that monocytes arriving from blood into a tissue are rapidly activated and polarised into M1 cells and, in a resolving inflammatory reaction, they later develop a classical M2 deactivated functional profile. Conversely, in a persistent inflammatory reaction, the incoming blood monocytes are activated into M1 cells, but with time they only partially maintain the M1 characteristics and concomitantly develop a typical M2 deactivated phenotype. These cells with a mixed M1-M2 phenotype may contribute to the later sequelae of persistent inflammation, in which the M1-like cell- and tissue-destructive activities are paralleled by M2-like anti-inflammatory and tissue-remodeling effects. This also shows that a persistent inflammatory stimulation cannot maintain the M1 phenotype of incoming monocytes for long, suggesting that the long-term presence of M1 cells and effects in a chronically inflamed tissue is mainly due to activation of newly incoming cells.

Monocytes and macrophages have different roles in inflammation. Monocytes are highly active in producing inflammatory cytokines and act as direct effectors (see below). Conversely, tissue-resident macrophages are more important in maintaining tissue homeostasis and surveillance, and, in an inflammatory reaction, in recruiting effector cells (by producing chemokines) and later in repairing/remodeling the damaged tissue. Observations in our *in vitro* models are in agreement with these notions, as we can see that surviving cells are less active in producing inflammatory cytokines, while they differentiate into macrophages and acquire an M2 phenotypes. From transcriptomic analysis (Supplementary Figures 1, 2) we observe that M2-specific genes that remain up-regulated in the late phase of the inflammation include several chemokines (Supplementary Table 2) and, from pathway analysis, we confirm an enrichment of genes involved in chemotaxis and extracellular matrix organization, both peculiar functions of M2 macrophages.

We checked the expression level for nitric oxide synthases (NOS1, NOS2, and NOS3) and arginases (ARG1 and ARG2) as M1 and M2 markers, respectively. Only ARG2 was expressed at measurable levels, but we did not observe significant changes in its expression, either between different time points within each of the two models, or in the P96 vs. R48 or P24 vs. R24 comparisons (data not shown). This is in agreement with previous data. In fact, while nitric oxide synthase and arginase are measurable *in vitro* in murine macrophages and are M1 and M2 markers, their expression and activity in human macrophages *in vitro* is still controversial and debated (52–54).

Newly recruited monocytes can die during inflammation (as we observed in our models, especially in the persistent one), and the extent of their survival possibly depends on the nature and magnitude of the insult. However, we suppose that surviving cells (“monocyte-derived macrophages”), after initial recognition of microbial or damage-associated molecules, may become “memory macrophages”, i.e., cells functionally programmed by a previous stimulus for either enhanced (potentiation/training) or decreased (tolerance) reactivity to a subsequent related or unrelated challenge (55, 56). Results showing a shift in inflammasome genes in cells that have experienced different inflammatory microenvironments seem to suggest that this is possible. Cells surviving after resolution of an acute bacterial inflammation (as in the resolving model) displayed a gene expression pattern that privileges NLRC4-dependent inflammasome activation. Conversely, cells surviving in a persistently inflamed microevironment preferentially expressed AIM2 inflammasome genes. This would suggest that these cells have memory of the past challenges and have changed their reactivity in different directions, being more ready to react to intracellular bacteria [recognised by the NLRC4 inflammasome; (48)] in one case, and more susceptible to reacting to self-DNA [recognised by the AIM2 inflammasome; (49)] in the case of persistent inflammation.

In describing the functions of monocytes newly recruited in an inflamed tissue, we have focussed on the expression and production of IL-1 family of cytokines and receptors, because of the effector role of these factors in different phases of inflammation, and their cross-regulation along the course of inflammation, anti-inflammation/resolution and re-establishment of homeostasis (23, 26).

The main inflammatory cytokine of the family is IL-1β that, as well as that of its sister cytokine IL-1α, is tightly regulated to limit its powerful inflammatory potential (57). IL-1 binds to the receptor IL-1R1, which forms a signalling complex with the accessory chain IL-1R3 (58). The antagonist IL-1Ra also binds IL-1R1, competing with agonist IL-1 ligands and preventing the formation of the signalling complex (58). The extracellular IL-1R1 domain (sIL-1R1) can capture IL-1 in solution and inhibit its binding to the membrane receptors, but it can also bind IL-1Ra (58). The second receptor for IL-1, IL-1R2, binds IL-1β with high affinity and recruits IL-1R3, but does not initiate intracellular signalling, thereby acting as a decoy receptor (58). The soluble form of IL-1R2 preferentially binds IL-1β with high affinity, and is therefore the most powerful soluble receptor inhibitor of IL-1β (59). The affinity of sIL-1R2 for IL-1β is increased by the formation of trimeric soluble complexes with sIL-1R3 (58). IL-1β, IL-1Ra and soluble receptors are mainly released between 4 and 14 h during the course of both resolving and persistent inflammation *in vitro*. The free and biologically active IL-1β, calculated by considering the simultaneous levels of IL-1β, sIL-1R2, sIL-1R3, and IL-1Ra, is mainly present at 14 h, i.e., at the stage of full inflammation. Notably, the levels of free active IL-1β decrease with time both in the resolving and in the persistent inflammatory response, suggesting that stimulus-induced IL-1 release/activity is a characteristic feature of monocytes, which decreases with maturation of monocytes to macrophages, as it occurs in a tissue and also *in vitro*, independently of the persistent presence of the triggering stimulus (60, 61).

Another important member of the IL-1 family cytokines is IL-18, for which both inflammatory and metabolic/immunological regulatory effects have been described (62). IL-18 activity is inhibited by IL-18BP, a soluble receptor-like protein able to bind IL-18 with high affinity thereby preventing its activating interaction with membrane receptors (63). IL-18 induces the production of IFN-γ, which is the most potent stimulus for the production of IL-18BP, thereby self-regulating its own activity through the indirect induction of its inhibitor (63, 64). In the two *in vitro* models the rate of IL-18 production is high at 4 and 14 h (early and full inflammation), while the production of IL-18BP peaks at 4 h (early inflammation), then decreases and comes up again at the latest time points of persistent inflammation (72 and 96 h). The kinetics of free IL-18 (not bound by its inhibitor IL-18BP) fully overlaps that of total IL-18.

The increase of IL-18 and IL-1β during the inflammatory phases and their decrease with the progress of the reaction toward resolution is expected in a normal inflammatory response, in which the inflammatory factors must be depleted or neutralised after the elimination of the pathogen. In parallel, the increase of the IL-18 inhibitor IL-18BP and the presence of the IL-1 inhibitors IL-1Ra and sIL-1R2 respond to the same need to turn off the acute reaction to proceed to the stage of restoration of homeostasis. The observed kinetics of *IL1B, IL18, IL18BP, IL1RN*, and *IL1R2* expression also reflect the process of macrophage polarisation, which predicts low levels of IL-1β and IL-18 (inflammatory cytokines) and sustained levels of IL-1Ra, sIL-1R2, and IL-18BP (anti-inflammatory mediators) in M2 macrophages and the opposite trend in M1 cells (13, 65). It should be noted that the release of sIL-1R2 from monocytes involves the regulated cleavage of the membrane receptor by a specific protease (66). Thus, the rate of sIL-1R2 release does not necessarily correlate with the up-regulation of the *IL1R2* gene expression, but it reflects the need of extending IL-1 inhibition beyond the immediate cell microenvironment during acute inflammation (highest rate of sIL-1R2 release in the absence of gene up-regulation), while late gene up-regulation during resolution, accompanied by limited sIL-1R2 release, would underline the down-regulation of IL-1 activity at the cellular level for re-establishing homeostasis. The rate of soluble inhibitors' production represents the inhibition of the paracrine IL-1 effects and contributes to limit the extent of the inflammatory reaction.

Looking at RNAseq signals, it is interesting to note that *SIGIRR*, the gene coding for the anti-inflammatory orphan receptor IL-1R8, is expressed in fresh monocytes and it is strongly down-regulated during inflammation, to increase again during resolution and the persistence of inflammation. This is what we expect for an anti-inflammatory gene, however the IL-1R8 protein is not present in monocytes, either on the cell membrane or intracellularly (data not shown). While this observation confirms that monocytes do not use IL-1R8 for down-regulating inflammation (67, 68), the inflammation-dependent modulation of its transcription suggests a different role for these transcripts in the regulation of monocyte/macrophage inflammatory pathways.

It is notable that in the model of persistent inflammation many of the changes observed in resolving inflammation are maintained. Thus, the release of inflammatory factors such as IL-1β and IL-18 decreases despite the persistence of inflammatory stimuli, and anti-inflammatory factors increase. The latter event is expected, as it is known that in chronic persistent inflammation the anti-inflammatory factors are actually produced at higher rates, in the attempt to down-regulate persistently present inflammatory factors (69).

Overall, concentrations of secreted IL-1 family members gradually increased, peaked between 4 and 14 h and decreased thereafter. This acute release during the early phase of resolving or persistent inflammatory reactions may represent the initiation of the cascade of inflammatory responses and subsequent adaptive immune responses (26). Indeed, IL-1β, IL-18, and IL-33 also contribute to the host defence against infections by regulating Th17, Th1, and Th2 CD4 T cell responses (25).

Moreover, it has long been known that IL-1 (both IL-1α and IL-1β) drives haematopoiesis, by increasing myeloid cell production and their release from the bone marrow in response to infection and inflammation (70–72). While IL-1 is not involved in steady-state haematopoiesis, it drives emergency haematopoiesis, by accelerating stem cell differentiation and promoting myeloid differentiation through induction of the myeloid transcriptional regulator PU.1 (73). Thus, the ability of IL-1 to induce the release of myeloid cells from bone marrow, in particular monocytes, appears as an important mechanism in the persistence of M1-mediated inflammation, supporting the hypothesis of a continuous influx of new effector monocytes from the bone marrow to the inflammatory site.

In conclusion, we have observed some important aspects in the evolution of monocyte activation during inflammation. In the late phase of the resolving inflammatory response monocytes resemble M2-like macrophages, while in the late phase of persistent inflammation they acquire a mixed M1/M2-like phenotype. In agreement with their expected functional differences, monocyte-derived macrophages during the resolution phase of inflammation are molecularly very different from monocyte-derived macrophages that are surviving in a persistently inflamed microenvironment. This is underlined by the fact that their capacity to produce IL-1β and IL-18 apparently depends on different inflammasomes and can be therefore activated by different types of triggering events. Overall, differentiated macrophages have decreased capacity to produce stimulus-induced inflammatory factors as compared to fresh monocytes (see levels of IL-1β in Figure 7 and in Supplementary Figure 3). Thus, we hypothesize that the production of inflammatory mediators during an *in vivo* inflammation is mainly due to newly recruited blood monocytes, and that the well-described role of IL-1 family cytokines and receptors in chronic inflammation is most likely dependent on the continuous influx of blood monocytes into a chronically inflamed site. On the other hand, monocyte-derived macrophages that have survived an inflammatory reaction can become memory cells that have a capacity to react to stimuli that is different from that of monocytes and different depending on the type of inflammatory event they experienced. The preliminary data shown here on the different inflammasome reactivity of macrophages after a resolved inflammation vs. a persistent reaction suggests that memory could bias the response of macrophages either toward more effective protection or toward pathological reactions.

## Data Availability Statement

The datasets generated for this study can be found in the ArrayExpress database at EMBL-EBI (www.ebi.ac.uk/arrayexpress) under accession number E-MTAB-8226.

## Ethics Statement

The ongoing study on human monocyte activation was approved by the Ethical Committee of the University of Pisa S. Chiara Hospital (prot. AOUP 33998 of September 29, 2006). All samples of human blood included in this study were from anonymous donors after informed consent. The patients/participants provided their written informed consent to participate in this study.

## Author Contributions

PI designed the study, performed the experiments, and wrote the paper. EM performed the bioinformatics analysis. GD and DM performed ELISA experiments. PM designed the study and revised the manuscript. LM revised the manuscript. DB designed the study and revised the manuscript. All authors contributed to the article and approved the submitted version.

## Conflict of Interest

The authors declare that the research was conducted in the absence of any commercial or financial relationships that could be construed as a potential conflict of interest.
