# Supplementary material for: Profiling the Course of Resolving vs. Persistent Inflammation in Human Monocytes: The Role of IL-1 Family Molecules
Source: Front Immunol. 2020 Jul 10;11:1426. doi: 10.3389/fimmu.2020.01426 (PMC7365847; doi:10.3389/fimmu.2020.01426)
Supplement: Supplementary file 7 [file Data_Sheet_1.PDF]

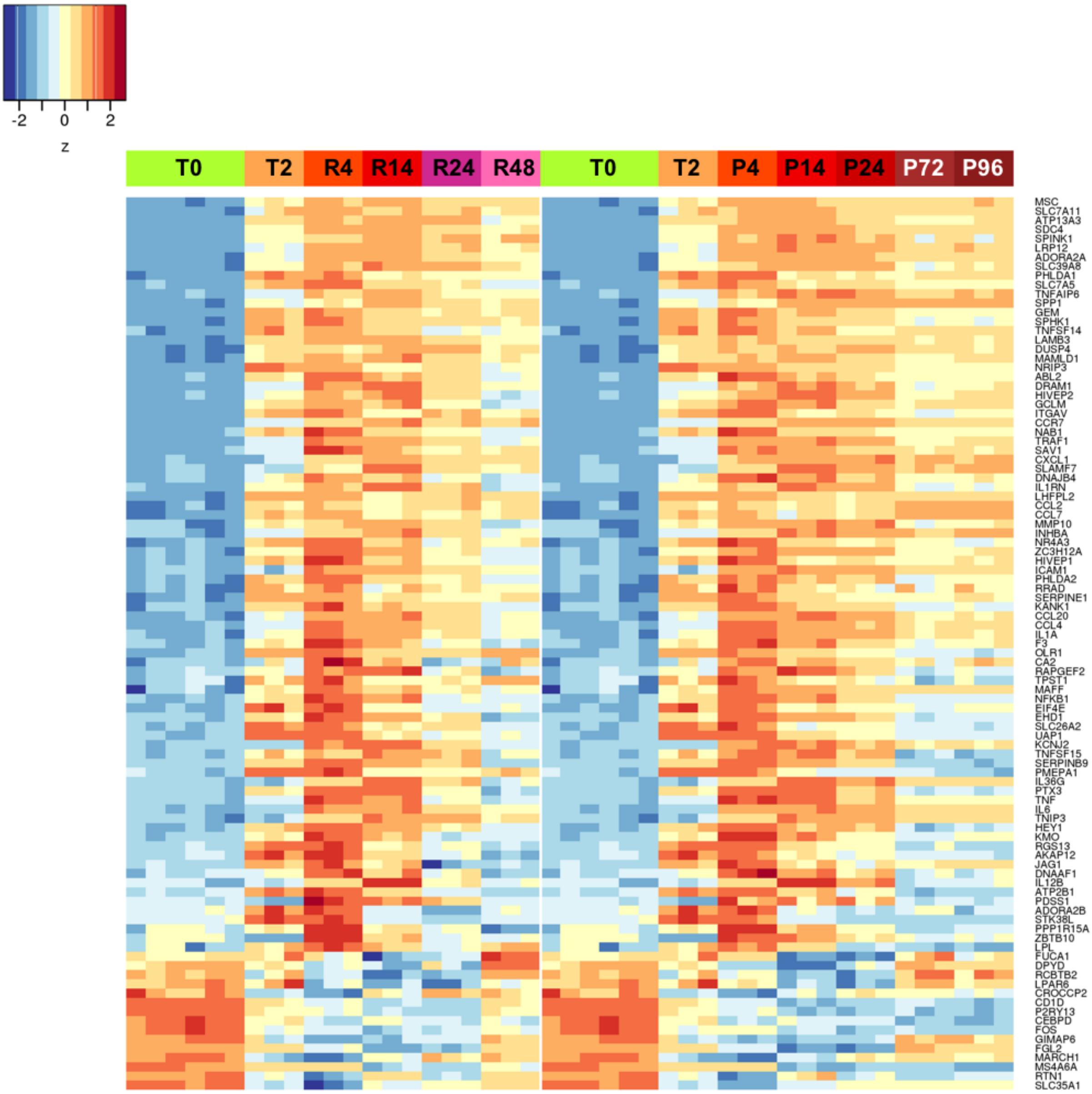

Figure S1. Differentially expressed genes in M1 macrophages *vs.* fresh monocytes.
The gene list originates from a meta-database with genes identified by SAM as statistically down-regulated (blue) or up-regulated (red) in M1 samples compared to fresh unstimulated monocytes, excluding genes that are modulated in M2 *vs.* fresh monocytes (45). This list was used to cluster samples of the two kinetic models of resolving and persistent inflammation. Heat-maps represent fold-expression levels of the monocyte-to-M1 genes assessed in the *in vitro* models of resolving inflammation (left) and persistent inflammation (right). Time points are indicated as T0 (fresh monocytes), T2 (after 2 h with CCL2, common to both models), R4 and P4 (4 h of resolving and persistent models), R14 and P14 (14 h both models), R24 and P24 (24 h both models), R48 (48 h resolving model only), P72 and P96 (72 and 96 h persistent model only).

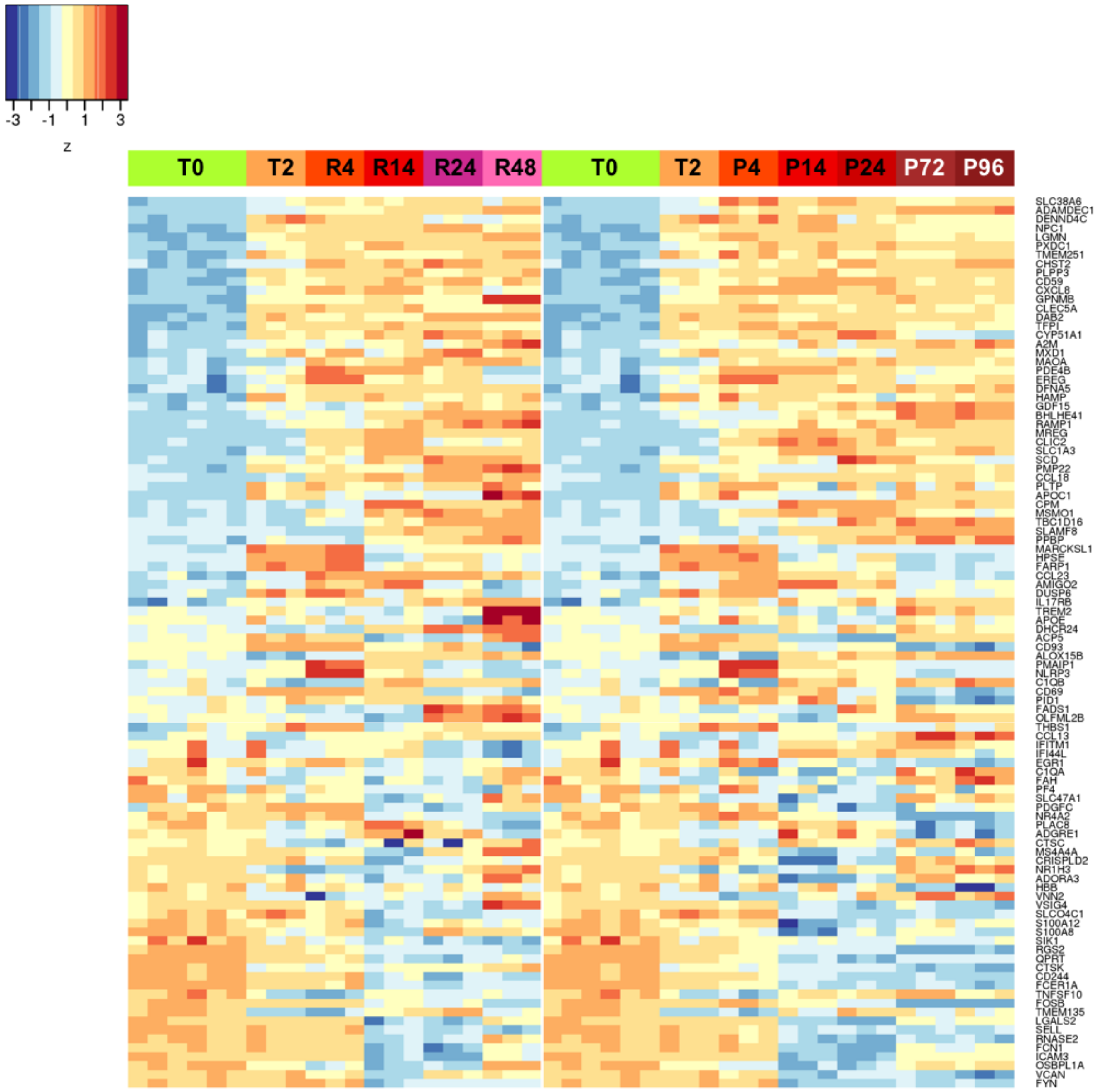

Figure S2. Differentially expressed genes in M2 macrophages *vs.* fresh monocytes.

The gene list originates from a meta-database with genes identified by SAM as statistically down-regulated (blue) or up-regulated (red) in M2 samples compared to fresh unstimulated monocytes, excluding genes that are modulated in M1 *vs.* fresh monocytes (45). This list was used to cluster samples of the two kinetic models of resolving and persistent inflammation. Heat-maps represent fold-expression levels of the monocyte-to-M2 genes assessed in the *in vitro* models of resolving inflammation (left) and persistent inflammation (right). Time points are indicated as described in the legend to Supplementary Figure S1.

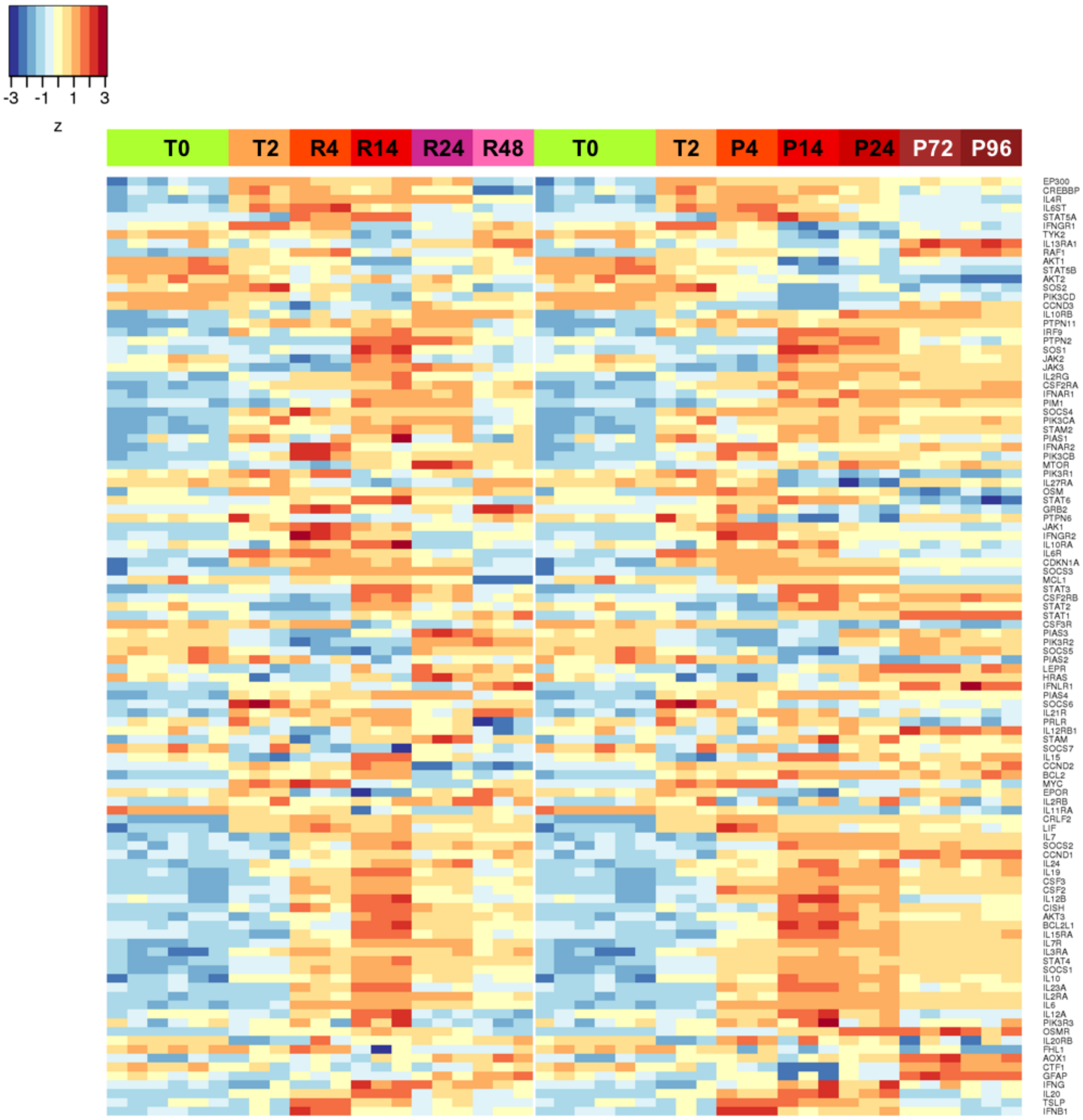

Figure S3. Differentially expressed genes of the JAK/STAT pathway during the different phases of in vitro resolving and persistent inflammation.

Heat-maps represent fold-expression levels of the genes involved in the JAK/STAT signalling pathway, assessed in the *in vitro* models of resolving (left) and persistent inflammation (right). The list of genes examined for JAK/STAT signalling is contained in https://www.ncbi.nlm.nih.gov/biosystems/?term=83077 . Time points are indicated as described in the legend to Supplementary Figure S1.

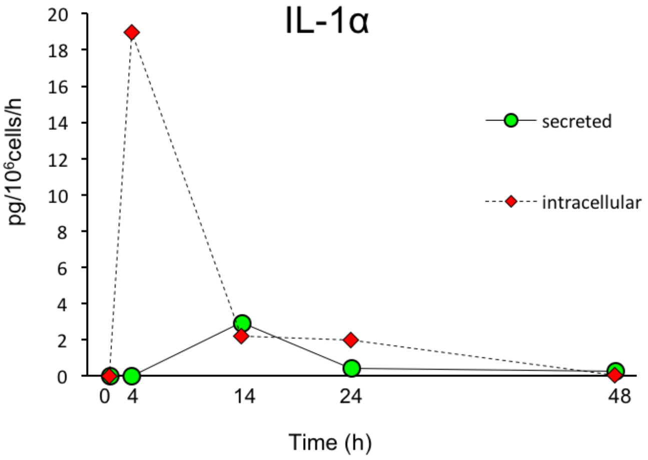

Figure S4. Intracellular and secreted IL-1α levels during an *in vitro* resolving inflammatory reaction.

Production of intracellular (green circles, continuous line) and secreted (red diamonds, dashed line) IL-1α during the resolving *in vitro* inflammatory reaction. The levels of the protein released in the supernatant or present in the cell lysate is reported in terms of rate of production, *i.e.*, the amount of protein produced per million viable cells per hour. The average values of one representative donor are reported. SD are not visible in the figure.

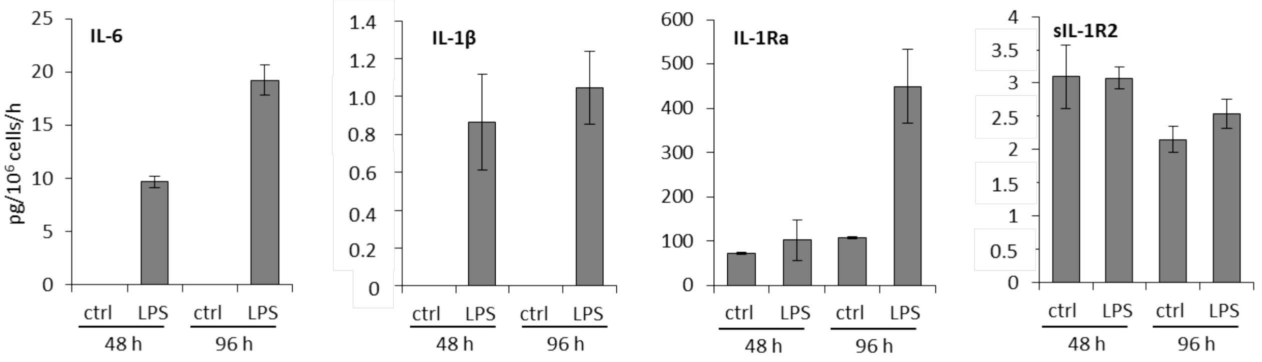

Figure S5. Reactivity to inflammatory stimulation of monocytes kept in culture for 48 and 96 h.

Freshly isolated human blood monocytes were kept in culture for 48 h and 96 h and then stimulated with LPS (10 ng/ml) for 24 h. The production of IL-6 and IL-1 family cytokines released in the supernatant is reported in terms of rate of production, *i.e.*, the amount of protein produced per million viable cells per hour. The mean values of triplicate determination from cells of one donor are reported. Statistical significance was calculated with one-way ANOVA followed by Tukey's multiple comparisons test. A *P* value <0.05 was considered statistically significant. IL-6 ctrl *vs*. LPS (at 48 and 96 h) *P*<.0001; IL-6 LPS 48 *vs*. 96 h *P*<.0001; IL-1β ctrl *vs*. LPS (at 48 and 96 h) *P*<.001; IL-1Ra ctrl *vs*. LPS (at 96 h) *P*<.0001; IL-1Ra LPS 48 *vs*. 96 h *P*<.0001; sIL-1R2 ctrl 48 *vs*. 96 h *P*<.05; sIL-1R2 LPS 48 *vs*. 96 h *P*<.05.
