# Supplementary material for: Profiling the Course of Resolving vs. Persistent Inflammation in Human Monocytes: The Role of IL-1 Family Molecules
Source: Front Immunol. 2020 Jul 10;11:1426. doi: 10.3389/fimmu.2020.01426 (PMC7365847; doi:10.3389/fimmu.2020.01426)
Supplement: Supplementary file 8 [file Data_Sheet_2.PDF]

Table S7. One-way ANOVA and Tukey's multiple comparisons test on time point ranges in the resolving and persistent models.

**IL-1α**	**2-4 h**	**4-14 h**	**14-24 h**	**24-48 h**	**24-72 h**	**72-96 h**	**24-96 h**
Resolving	ns	ns	ns	ns	-	-	-
Persistent	ns	ns	ns	-	ns	ns	Ns
**IL-1β**	**2-4 h**	**4-14 h**	**14-24 h**	**24-48 h**	**24-72 h**	**72-96 h**	**24-96 h**
Resolving	ns	< .0001	ns	0.0416	-	-	-
Persistent	ns	< .0001	< .0001	-	ns	ns	ns
**IL-1Ra**	**2-4 h**	**4-14 h**	**14-24 h**	**24-48 h**	**24-72 h**	**72-96 h**	**24-96 h**
Resolving	0.0002	ns	ns	ns	-	-	-
Persistent	ns	ns	0.0015	-	ns	ns	Ns
**sIL-1R1**	**2-4 h**	**4-14 h**	**14-24 h**	**24-48 h**	**24-72 h**	**72-96 h**	**24-96 h**
Resolving	< .0001	< .0001	ns	ns	-	-	-
Persistent	< .0001	< .0001	< .0001	-	ns	ns	0.0047
**sIL-1R2**	**2-4 h**	**4-14 h**	**14-24 h**	**24-48 h**	**24-72 h**	**72-96 h**	**24-96 h**
Resolving	< .0001	< .0001	ns	ns	-	-	-
Persistent	< .0001	< .0001	< .0001	-	ns	ns	0.0014
**sIL-1R3**	**2-4 h**	**4-14 h**	**14-24 h**	**24-48 h**	**24-72 h**	**72-96 h**	**24-96 h**
Resolving	< .0001	< .0001	ns	ns	-	-	-
Persistent	< .0001	< .0001	< .0001	-	0.0212	0.0119	< .0001
**Free IL-1β**	**2-4 h**	**4-14 h**	**14-24 h**	**24-48 h**	**24-72 h**	**72-96 h**	**24-96 h**
Resolving	ns	< .0001	ns	0.0426	-	-	-
Persistent	ns	< .0001	< .0001	-	ns	ns	ns
**Active IL-1β**	**2-4 h**	**4-14 h**	**14-24 h**	**24-48 h**	**24-72 h**	**72-96 h**	**24-96 h**
Resolving	ns	< .0001	0.0095	ns	-	-	-
Persistent	ns	0.0005	0.0006	-	ns	ns	ns
**IL-18**	**2-4 h**	**4-14 h**	**14-24 h**	**24-48 h**	**24-72 h**	**72-96 h**	**24-96 h**
Resolving	ns	ns	0.0254	ns	-	-	-
Persistent	0.0006	ns	0.0004	-	ns	ns	ns
**IL-18BP**	**2-4 h**	**4-14 h**	**14-24 h**	**24-48 h**	**24-72 h**	**72-96 h**	**24-96 h**
Resolving	< .0001	< .0001	ns	ns	-	-	-
Persistent	0.0002	0.0169	ns	-	ns	ns	ns
**Free IL-18**	**2-4 h**	**4-14 h**	**14-24 h**	**24-48 h**	**24-72 h**	**72-96 h**	**24-96 h**
Resolving	ns	ns	ns	ns	-	-	-
Persistent	0.0006	ns	0.0281	-	ns	ns	ns
**IL-36β**	**2-4 h**	**4-14 h**	**14-24 h**	**24-48 h**	**24-72 h**	**72-96 h**	**24-96 h**
Resolving	< .0001	< .0001	ns	ns	-	-	-
Persistent	< .0001	< .0001	0.0022	-	ns	ns	ns
**IL-36γ**	**2-4 h**	**4-14 h**	**14-24 h**	**24-48 h**	**24-72 h**	**72-96 h**	**24-96 h**
Resolving	< .0001	< .0001	ns	ns	-	-	-
Persistent	< .0001	< .0001	ns	-	ns	ns	ns

A P value <0.05 was considered statistically significant.
ns: not significant; -: not applicable.

Table S8. **One-way ANOVA and Tukey's multiple comparisons test on single time point in the resolving and persistent models (R *vs.* P).**

**IL-1α**	R2h	R4h	R14h	R24h	R48h		**IL-1β**	R2h	R4h	R14h	R24h	R48h
P2h	ns	ns	ns	ns	ns		P2h	ns	ns	<.0001	0.0054	ns
P4h	ns	ns	ns	ns	ns		P4h	ns	ns	<.0001	0.0103	ns
P14h	ns	ns	ns	ns	ns		P14h	<.0001	<.0001	ns	0.0006	<.0001
P24h	ns	ns	ns	ns	ns		P24h	ns	ns	<.0001	0.0065	ns
P72h	ns	ns	ns	ns	ns		P72h	ns	ns	<.0001	0.0344	ns
P96h	ns	ns	ns	ns	ns		P96h	ns	ns	<.0001	0.0070	ns
**IL-1Ra**	R2h	R4h	R14h	R24h	R48h		**sIL-1R1**	R2h	R4h	R14h	R24h	R48h
P2h	ns	0.0003	ns	0.0067	ns		P2h	ns	<.0001	ns	ns	0.0108
P4h	ns	0.0295	ns	ns	ns		P4h	<.0001	ns	<.0001	<.0001	<.0001
P14h	0.0011	ns	ns	ns	ns		P14h	<.0001	<.0001	<.0001	<.0001	<.0001
P24h	ns	0.0003	ns	0.0068	ns		P24h	ns	<.0001	ns	ns	ns
P72h	ns	0.0056	ns	ns	ns		P72h	ns	<.0001	ns	ns	ns
P96h	ns	0.0035	ns	ns	ns		P96h	ns	<.0001	ns	ns	0.0016
**sIL-1R2**	R2h	R4h	R14h	R24h	R48h		**sIL-1R3**	R2h	R4h	R14h	R24h	R48h
P2h	ns	<.0001	ns	ns	ns		P2h	<.0001	ns	<.0001	<.0001	<.0001
P4h	<.0001	<.0001	<.0001	<.0001	<.0001		P4h	<.0001	<.0001	<.0001	<.0001	<.0001
P14h	0.0174	<.0001	<.0001	0.0003	<.0001		P14h	<.0001	ns	<.0001	<.0001	<.0001
P24h	0.0343	<.0001	ns	ns	ns		P24h	ns	<.0001	ns	ns	ns
P72h	ns	<.0001	ns	ns	ns		P72h	0.0289	<.0001	ns	0.0051	0.0019
P96h	ns	<.0001	0.0124	ns	0.0025		P96h	<.0001	ns	<.0001	<.0001	<.0001
**Free IL-1β**	R2h	R4h	R14h	R24h	R48h		**Active IL-1β**	R2h	R4h	R14h	R24h	R48h
P2h	ns	ns	<.0001	0.0056	ns		P2h	ns	ns	<.0001	ns	ns
P4h	ns	ns	<.0001	0.0090	ns		P4h	ns	ns	<.0001	ns	ns
P14h	<.0001	<.0001	ns	0.0007	<.0001		P14h	0.0006	0.0003	ns	ns	0.0070
P24h	ns	ns	<.0001	0.0067	ns		P24h	ns	ns	<.0001	ns	ns
P72h	ns	ns	<.0001	0.0348	ns		P72h	ns	ns	0.0003	ns	ns
P96h	ns	ns	<.0001	0.0071	ns		P96h	ns	ns	<.0001	ns	ns

A P value <0.05 was considered statistically significant.
ns: not significant; in yellow the statistical significance discussed in the manuscript.

Table S9. **One-way ANOVA and Tukey's multiple comparisons test on single time point in the resolving and persistent models.**

**IL-18**	R2h	R4h	R14h	R24h	R48h
P2h	ns	ns	ns	ns	ns
P4h	0.0006	ns	ns	0.0001	<.0001
P14h	0.0031	ns	ns	0.0007	0.0003
P24h	ns	ns	0.0171	ns	ns
P72h	ns	ns	0.0194	ns	ns
P96h	ns	ns	0.030	ns	ns

**IL-18BP**	R2h	R4h	R14h	R24h	R48h
P2h	ns	<.0001	ns	ns	ns
P4h	0.0002	ns	0.0001	0.0002	<.0001
P14h	ns	0.0001	ns	ns	ns
P24h	ns	<.0001	ns	ns	ns
P72h	ns	0.0026	ns	ns	0.0334
P96h	ns	0.0032	ns	ns	0.0273

**Free IL-18**	R2h	R4h	R14h	R24h	R48h
P2h	ns	ns	ns	ns	ns
P4h	0.0006	ns	ns	0.0001	<.0001
P14h	ns	ns	ns	0.0414	0.0197
P24h	ns	ns	ns	ns	ns
P72h	ns	ns	ns	ns	ns
P96h	ns	ns	ns	ns	ns

**IL-36β**	R2h	R4h	R14h	R24h	R48h
P2h	ns	<.0001	ns	ns	ns
P4h	<.0001	ns	<.0001	<.0001	<.0001
P14h	ns	<.0001	0.0021	0.0084	0.0038
P24h	ns	<.0001	ns	ns	ns
P72h	ns	<.0001	ns	ns	ns
P96h	ns	<.0001	ns	ns	ns

**IL-36γ**	R2h	R4h	R14h	R24h	R48h
P2h	ns	<.0001	ns	ns	ns
P4h	<.0001	ns	<.0001	<.0001	<.0001
P14h	ns	0.0012	ns	ns	ns
P24h	ns	<.0001	ns	ns	ns
P72h	ns	<.0001	ns	ns	ns
P96h	ns	0.0004	ns	ns	ns

A P value <0.05 was considered statistically significant.

ns: not significant; in yellow the statistical significance discussed in the manuscript.
